# Performance of a deep convolutional neural network for MRI-based vertebral body measurements and insufficiency fracture detection

**DOI:** 10.1007/s00330-022-09354-6

**Published:** 2022-12-28

**Authors:** Christoph Germann, André N. Meyer, Matthias Staib, Reto Sutter, Benjamin Fritz

**Affiliations:** 1 Department of Radiology, Balgrist University Hospital, Forchstrasse 340, 8008 Zurich, Switzerland; 2 Faculty of Medicine, University of Zurich, Zurich, Switzerland; 3 ScanDiags AG, Zurich, Switzerland

**Keywords:** Artificial intelligence, Neural networks (computer), Magnetic resonance imaging, Spinal fractures

## Abstract

**Objectives:**

The aim is to validate the performance of a deep convolutional neural network (DCNN) for vertebral body measurements and insufficiency fracture detection on lumbar spine MRI.

**Methods:**

This retrospective analysis included 1000 vertebral bodies in 200 patients (age 75.2 ± 9.8 years) who underwent lumbar spine MRI at multiple institutions. 160/200 patients had ≥ one vertebral body insufficiency fracture, 40/200 had no fracture. The performance of the DCNN and that of two fellowship-trained musculoskeletal radiologists in vertebral body measurements (anterior/posterior height, extent of endplate concavity, vertebral angle) and evaluation for insufficiency fractures were compared. Statistics included (a) interobserver reliability metrics using intraclass correlation coefficient (ICC), kappa statistics, and Bland-Altman analysis, and (b) diagnostic performance metrics (sensitivity, specificity, accuracy). A statistically significant difference was accepted if the 95% confidence intervals did not overlap.

**Results:**

The inter-reader agreement between radiologists and the DCNN was excellent for vertebral body measurements, with ICC values of > 0.94 for anterior and posterior vertebral height and vertebral angle, and good to excellent for superior and inferior endplate concavity with ICC values of 0.79–0.85. The performance of the DCNN in fracture detection yielded a sensitivity of 0.941 (0.903–0.968), specificity of 0.969 (0.954–0.980), and accuracy of 0.962 (0.948–0.973). The diagnostic performance of the DCNN was independent of the radiological institution (accuracy 0.964 vs. 0.960), type of MRI scanner (accuracy 0.957 vs. 0.964), and magnetic field strength (accuracy 0.966 vs. 0.957).

**Conclusions:**

A DCNN can achieve high diagnostic performance in vertebral body measurements and insufficiency fracture detection on heterogeneous lumbar spine MRI.

**Key Points:**

*• A DCNN has the potential for high diagnostic performance in measuring vertebral bodies and detecting insufficiency fractures of the lumbar spine.*

**Supplementary Information:**

The online version contains supplementary material available at 10.1007/s00330-022-09354-6.

## Introduction

Several studies have been investigating applications of artificial intelligence (AI) in musculoskeletal imaging [1–14]. Deep learning (DL) as a subset of machine learning can be used for various tasks such as image recognition [15], image reconstruction and image data transformation [16–18], tissue segmentation [19, 20], workflow support, and disease detection [15–17, 21].

Vertebral body fractures are a common pathology and frequently encountered in daily radiological practice. Two main categories of vertebral body fractures exist: traumatic and atraumatic. The latter can be further subclassified into (1) insufficiency fractures, e.g., in patients with osteoporosis, and (2) pathologic fractures caused by focal bone abnormalities, like neoplasia [22]. Insufficiency fractures of the vertebral bodies are typically compression fractures, characterized by the impression of at least one endplate [23]. Depending on the morphology of the fracture and involvement of the other endplate or the posterior wall, compression fractures can be classified into wedge, concave, or crush deformities [23]. In addition, the extent of vertebral height loss is of high clinical importance, which, combined with the compression fracture type, can influence treatment and rehabilitation [24–26]. Therefore, the detection and precise morphologic quantification of insufficiency fractures are crucial in clinical practice.

First-line imaging in patients with non-traumatic back pain comprises radiography with anterior-posterior and lateral views [27]. Further diagnostic workup frequently includes MR imaging, e.g., ruling out radiographically occult fractures, detecting neural compression, and guiding therapy. A few studies investigated machine learning approaches for fracture detection on radiography, dual-energy X-ray absorptiometry (DEXA), and CT [13, 28–31], showing in parts high diagnostic accuracies. Recent studies applied a DCNN to distinguish between benign and malignant fractures and between fresh and old osteoporotic fractures on lumbar spine MRI datasets, indicating high diagnostic accuracies [32–34]. Regarding AI-based lumbar spine measurements, several studies have been published [14, 35–37]. However, to our knowledge, the potential of a DL architecture for quantitative vertebral body assessment together with insufficiency fracture detection on MRI has not been investigated thus far.

Therefore, this study aimed to validate the diagnostic performance of a DCNN for MRI-based vertebral body measurements and detection of insufficiency fractures of the lumbar spine.

## Materials and methods

This retrospective cohort study was approved by the local ethics committee (Cantonal Ethics Committee, Zurich). All included subjects gave written informed consent to use former or future imaging data for research purposes.

### Study population

**Fig. 1:**
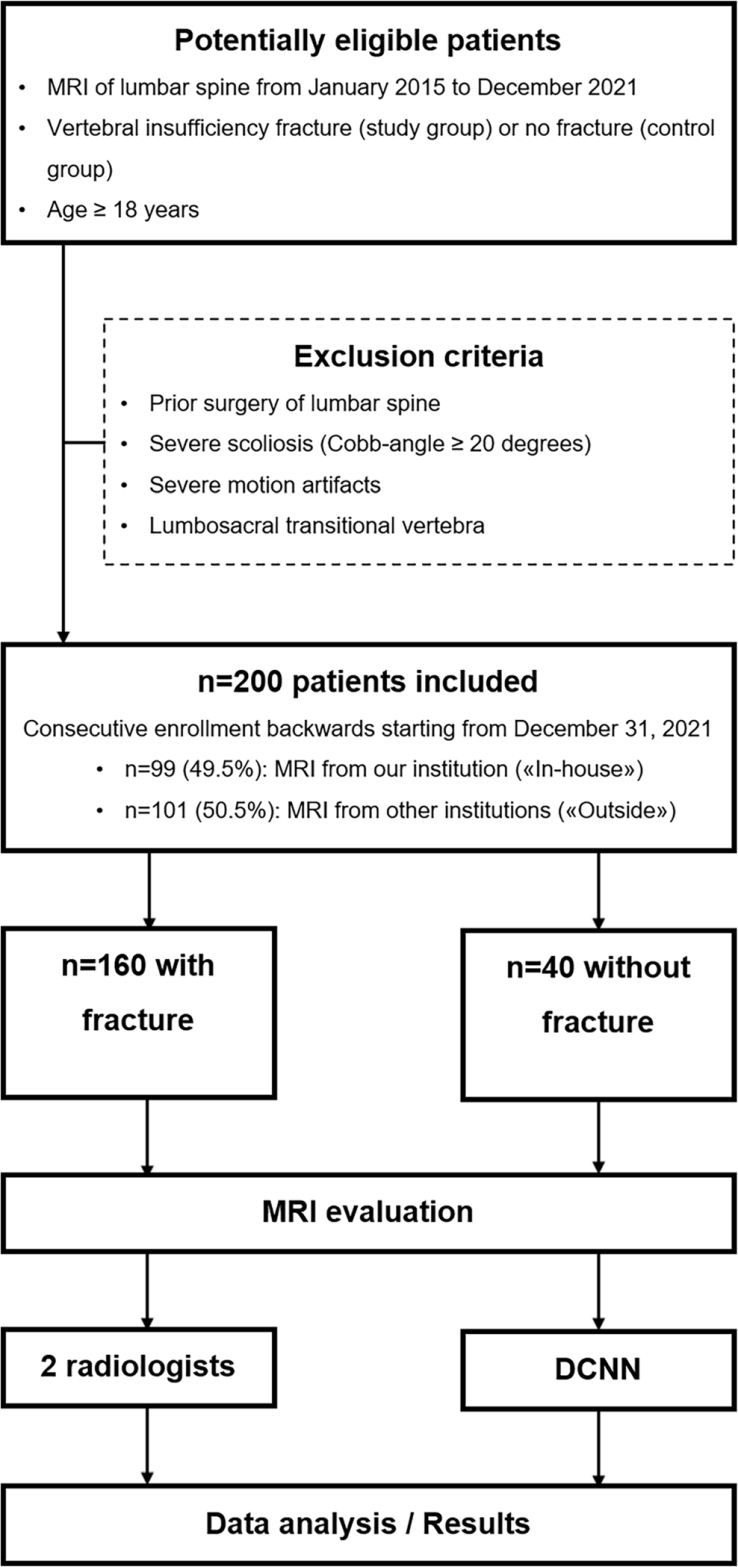
Flow chart of study design

We performed a database search for lumbar spine MRI. The first 160 patients (selected backwards and consecutively from December 2021) with vertebral insufficiency fractures were enrolled in the study. Forty consecutive patients without vertebral fractures served as the control group, resulting in a cohort size of *n* = 200. To test the DCNN with a heterogeneous cohort, we included examinations from our institution (“in-house”) and other hospitals/practices (“outside”) available in our picture archiving and communication system (PACS). The “outside” examinations comprised images from 12 different institutions: 44 examinations on GE, 56 examinations on Philips, and one examination on a Siemens scanner. A detailed flow chart of inclusion/exclusion is presented in Fig. 1.

### Magnetic resonance imaging

“In-house” MRI examinations were conducted on clinical 1.5-T or 3.0-T systems (MAGNETOM Avanto fit, MAGNETOM Skyra fit, MAGNETOM Vida, Siemens Healthcare GmbH) with varying imaging protocols as described in Table 1. MRI examinations in other institutions were performed on three major vendors’ clinical 1.5-T or 3.0-T scanners (GE Healthcare, Philips Healthcare, and Siemens Healthcare). To ensure adequate contrast properties and image quality, requirements of all included sagittal T2-weighted MR sequences were as follows: time of echo: ≥ 80 ms, time of repetition: ≥ 3000 ms, slice thickness: 2–4 mm, and in-plane voxel dimensions: ≤ 0.8 × 0.8 mm.

**Table 1:** MRI sequence parameter

Variable	Avanto fit 1.5 T	Skyra fit 3 T	Vida 3 T
Sequence type	T2w TSE	T2w TSE	T2w TSE
Imaging plane	Sagittal	Sagittal	Sagittal
TR, *ms*	4730	4000	3500
TE, *ms*	113	92	95
Refocusing flip angle, *degree*	150	130–150	160
FOV, *mm*	300	300	300
Pixel size, *mm*	0.7 × 0.7	0.7 × 0.7	0.6 × 0.6
Slices, *n*	15	15	15
Slice thickness, *mm*	4	4	4
Matrix	448 × 314	448 × 314	512 × 384
TA, *min:s*	2:01	0:53	0:56

Standard MR imaging protocol for the lumbar spine of the three scanner types included at our institution. Illustrated is the respective T2-weighted sagittal sequence, which was used for vertebral measurements and fracture detection

*FOV* field of view, *TA* acquisition time, *TE* echo time, *TR* repetition time

### MRI Interpretation

**Fig. 2:**
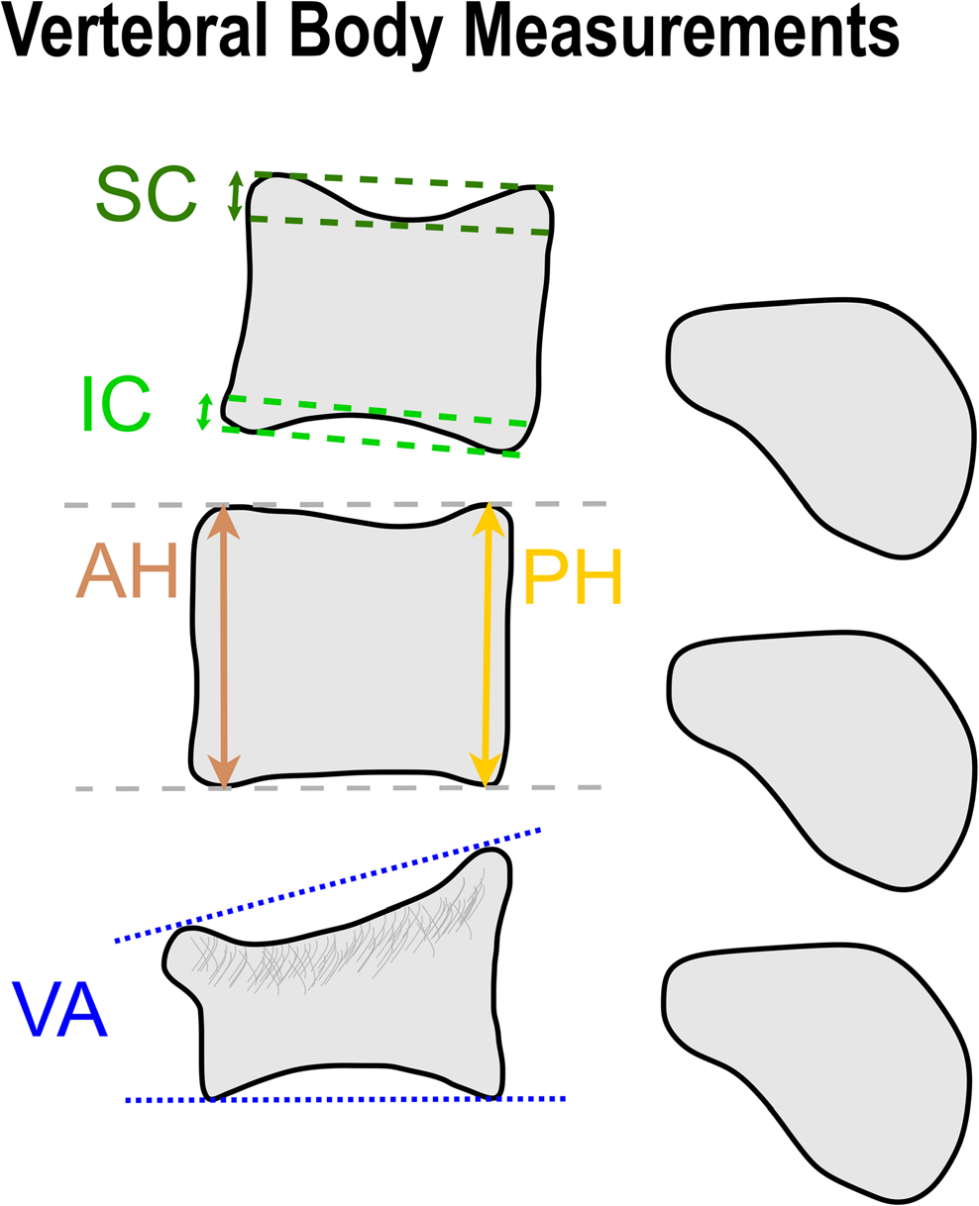
Schematic illustration of the vertebral body measurements. Anterior vertebral body height (AH) and posterior vertebral body height (PH) were defined as the maximum distance between each vertebral body’s superior and inferior endplate at the anterior or posterior aspect, respectively. The superior concavity (SC) and the inferior concavity (IC) were measured as the maximum distance of the depression of the superior endplate and inferior endplate, respectively. The vertebral angle (VA) is formed by the superior and inferior endplate (VA): a positive value signifies an anteriorly converging angle (as shown in this illustration), whereas a negative value corresponds to a posteriorly converging angle. *DCNN*, deep convolutional neural network

Two musculoskeletal radiologists evaluated each MRI independently (C.G. with 8 and B.F. with 10 years of experience). The image analysis comprised (a) measurements of vertebral body configuration and (b) classification of each vertebral body for the presence of insufficiency fractures. For the following measurements, each reader and the DCNN analyzed the same single sagittal slice through the middle of the vertebral bodies: (1) anterior vertebral body height (AH), (2) posterior vertebral body height (PH), (3 + 4) maximum extent of the concavity of the superior endplate (SC) and inferior endplate (IC), and (5) vertebral angle between superior and inferior endplates (VA) (Fig. 2). According to the Genant classification, a vertebral insufficiency fracture was defined as the presence of either a wedge, concave, or crush deformity, irrespective of fracture age [24]. A consensus reading was performed in case of disagreement between both readers regarding an insufficiency fracture. For this purpose, a third musculoskeletal radiologist (R.S. with 16 years of experience) resolved the disagreement.

### Deep convolutional neural network

The ground truth was created by medical annotators and radiologists on T2-weighted sagittal images using a medical annotation platform (https://www.trainingdata.io/). For each image, an annotation of the vertebrae was created, including upper sacral (S1), all lumbar (L1 to L5), and lower thoracic vertebrae (supplemental Figure 1).

#### Training, validation, and testing

The dataset for training, validation, and testing of the DCNN comprised 1056 lumbar spine MRIs from 2009 to 2019, originating from three different institutions using a clinical 1.5-T or 3.0-T MRI scanner. We used 834 of 1056 MRI datasets (79.0%) for training (i.e., compute the weights of the DCNN model), 104 of 1056 (9.8%) for validation (i.e., tune the parameters of the DCNN model), and 104 of 1056 (9.8%) for testing (i.e., assessing the model accuracy). We optimized the measurement algorithm on 37 studies (1.4%) included in the training set. The distribution between 1.5- and 3.0-T datasets was 83.6% vs. 16.4% in the training set, 82.7% vs. 17.3% in the validation set, and 79.8% vs. 20.2% in the testing set.

**Fig. 3:**
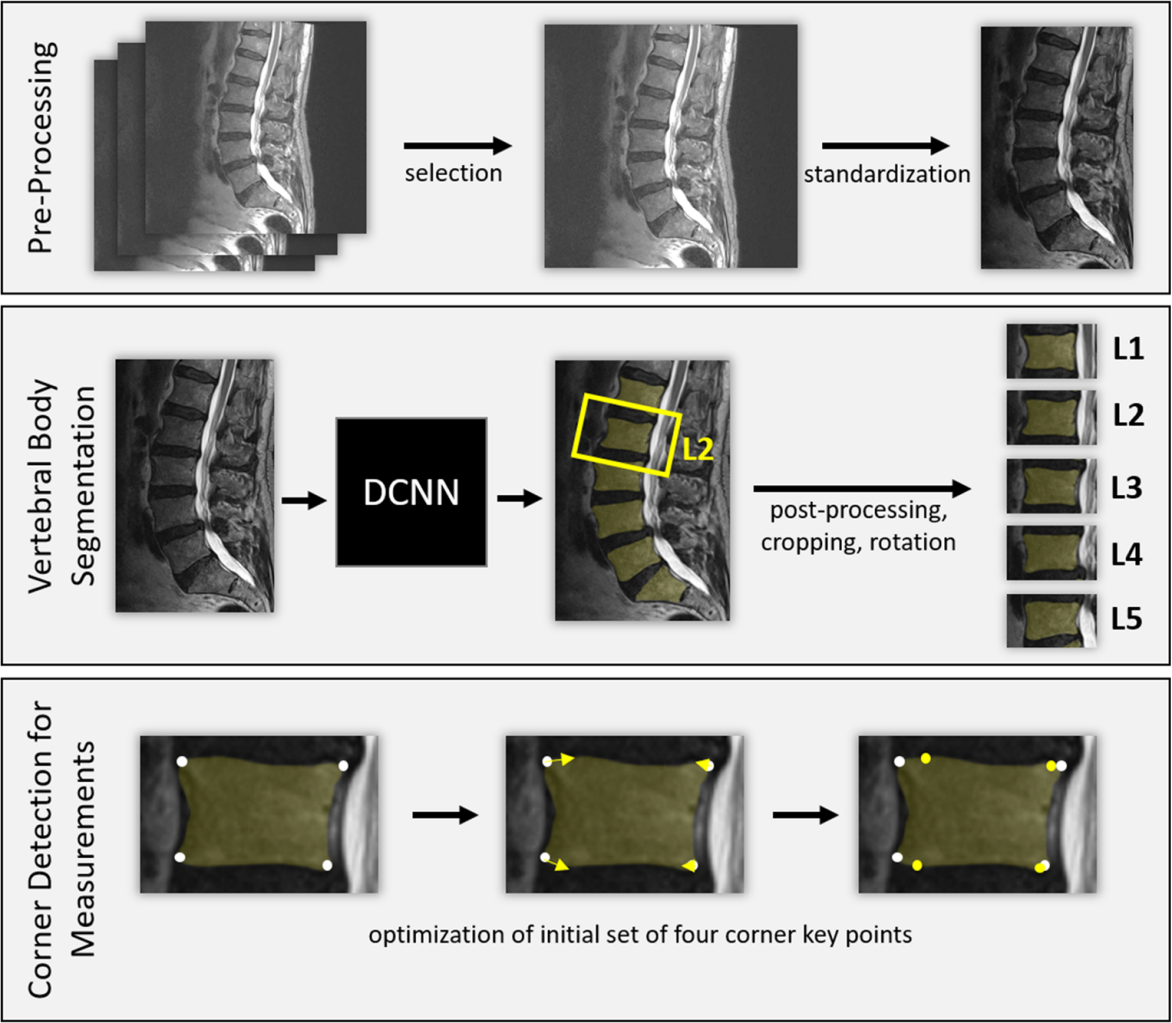
Illustration of the DCNN algorithm. First, in a pre-processing step, the center slice of the T2-weighted sagittal image stack was selected and standardized (resize, rescale shape, and intensity). Then, the lumbar vertebral bodies were segmented and annotated (L1 to L5). In a post-processing part, the image of each segmented vertebral body was cropped and rotated. Finally, the four corners of each vertebral body were identified and optimized according to the geometry. *DCNN*, deep convolutional neural network

The algorithm included three steps: first, image pre-processing and selection; second, vertebral body segmentation; and third, corner detection and measurements (Fig. 3).

#### Pre-processing

An algorithm selected the sagittal T2-weighted sequence from a lumbar spine MRI. The images were standardized by rescaling each image to 0.6 mm × 0.6 mm per voxel and resizing each slice to a matrix of 448 × 320. The MRI images were converted to 12bit (0 to 4096). During model development, the most informative intensity range to allow distinction between the vertebral body and anulus fibrosus was in the lower intensity range between 0 and 2500. Therefore, we opted to confine the algorithm to this range. Accordingly, voxel intensity values were divided by 2500 and clipped to 1.0, resulting in an intensity range of 0.0 to 1.0. All slices except the central one were discarded from further analysis. The center-left slice was used if a sequence contained an even number of slices.

#### Vertebral body segmentation

A DCNN performed image segmentation, consisting of an encoding path to capture the image context and a symmetric decoding path that produced a binary mask from a sequence of deconvolutions. The model follows the U-net architecture shown by Ronneberger et al [38]. The first four out of the five convolution blocks of the encoding path were concatenated to their mirrored decoding blocks (skip connections). Each convolution block was composed of two stacks of 3 × 3 convolution-, batch normalization-, and ReLU activation layers, followed by a 2 × 2 max pooling layer. The filter size for each block was increased by a factor of two, starting from 32 up to 512. The training task of the vertebra segmentation model was performed with a Dice coefficient loss function. We used the Adam algorithm as an optimizer for the learning process with a learning rate of 0.05. Finally, the TensorFlow (v2.2.0; https://tensorflow.org) backend was used to develop and train the DCNN. The training was performed on an NVIDIA Tesla V-100 graphics processing unit with a batch size of 16 and 100 epochs. The DCNN model was tested on 104 annotated images, resulting in a median Dice coefficient of 0.968, mean Dice coefficient of 0.964, and standard deviation of the Dice coefficient of 0.0167.

#### Corner detection and vertebral height measurement

The four corner points were defined for each vertebral body on the sagittal slice which served as anatomical landmarks for anterior height (AH), posterior height (PH), and vertebral angle (VA) measurements (Fig. 2). Specifically, we used three algorithms to determine these four anatomical landmarks: (1) the location of each point was optimized with a basin-hopping method starting from an initial set of points based on a bounding rectangle; (2) the curvature of the concave hull of the border of the vertebra segmentation was evaluated, and the four points with the highest curvature were extracted; and (3) straight lines along the vertebra border were defined and the corners were localized next to the intersections of these lines. Each strategy provided four vertebral corners. Of these three sets of corners, the one which maximized the overlap between the vertebral segmentation and a polygon spanned by the four corners was chosen.

##### Post-processing

Predicted vertebral segmentations underwent minor post-processing steps to remove anatomically implausible pixels by filling holes in the prediction, removing isolated pixel clusters that are too small, and removing pixel clusters that are located too far away from the majority of segmented vertebrae. For the visualization of individual vertebrae, the image was cropped and rotated around the center of each vertebra by a rotation angle derived from the alignment of the vertebrae.

#### Fracture detection

Using the vertebral body measurements mentioned above, the algorithm classified each vertebral body as either intact or fractured, according to the Genant classification [24].

A wedge deformity is present if the anterior ratio (anterior height divided by the posterior height) is three standard deviations lower than in a healthy population. A concave deformity is present if the ratio between the central height (mid vertebral body) and posterior height is three standard deviations below the reference ratio of a healthy population. A crush deformity signifies an overall height reduction of a vertebral body compared to its neighboring vertebra exceeding three standard deviations compared to a healthy population.

### Statistical analysis

Statistical analysis was performed using SPSS Version 25.0 (SPSS Statistics for Windows, IBM Corporation) and MedCalc version 17.6 (MedCalc Software bvba). Categorical data are presented as proportions and percentages, continuous data as means with standard deviation. Interobserver reliability was assessed by calculating the intraclass correlation coefficient (ICC) for vertebral body measurements and Cohen’s kappa for fracture detection. ICC values were interpreted according to Koo et al [39], kappa values according to Landis and Koch [40]. ICC values of both readers were averaged and subsequently compared to the DCNN assessments using Bland-Altman plots with limits of agreement (LoA) [41].

Diagnostic performance metrics (sensitivity, specificity, and accuracy) with 95% confidence intervals (CI) were calculated for the presence of fractures. A statistically significant difference was accepted if the 95% CI did not overlap.

## Results

### Study cohort

Table 2 presents detailed patient characteristics and scanner types. None of the included subjects was part of the training, validation, or testing set of the DCNN.

**Table 2:** Patient characteristics and scanner types

Variable	Entire cohort *n* = 200	With fracture *n* = 160	Without fracture *n* = 40
Age, *years*	75.2 ± 9.8	76.1 ± 9.7	71.7 ± 9.6
Gender, *n (%)*			
Male	75 (37.5)	52 (32.5)	23 (57.5)
Female	125 (62.5)	108 (67.5)	17 (42.5)
Fracture, *n (%)*			
L1	92 (54.0)	92 (57.5)	0 (0)
L2	52 (26.0)	52 (32.5)	0 (0)
L3	37 (18.5)	37 (23.1)	0 (0)
L4	32 (16.0)	32 (20.0)	0 (0)
L5	25 (12.5)	25 (15.6)	0 (0)
Institution, *n (%)*			
“In-house”	99 (49.5)	79 (49.4)	20 (50.0)
“Outside”	101 (50.5)	81 (50.6)	20 (50.0)
MRI vendor, *n (%)*			
Siemens	100 (50.0)	80 (50.0)	20 (50)
GE	44 (22.0)	33 (20.6)	11 (27.5)
Philips	56 (28.0)	47 (29.4)	9 (22.5)
MRI field strength, *n (%)*			
1.5 T	106 (53.0)	83 (51.9)	23 (57.5)
3.0 T	94 (47.0)	77 (48.1)	17 (42.5)

Demographic variables age, gender, vertebral insufficiency fracture, institution, MRI scanner vendor, and MRI field strength for (a) the entire cohort (*n* = 200), (b) the subgroup with vertebral body fracture (*n* = 160), and (c) the subgroup without any vertebral body fracture (*n* = 40). “In-house” refers to MRI scans conducted in our institution, and “outside” indicates acquisitions of other radiological institutions. Age is presented as mean ± standard deviation. All qualitative variables are given in numbers (percentages)

This study included 200 subjects: 160 subjects (80.0%) with at least one vertebral body insufficiency fracture in the lumbar spine and 40 subjects (20.0%) without any lumbar vertebral body fracture. The mean age in the cohort was 75.2 ± 9.8. Seventy-five of 200 patients (37.5%) were men, and 125 of 200 patients (62.5%) were women. Out of 1000 examined lumbar vertebral bodies, 238 (23.8%) had an insufficiency fracture and 762 vertebral bodies (76.2%) were intact, respectively. Insufficiency fractures occurred in 92 of 200 (46.0%) L1 vertebrae, 52 of 200 (26.0%) L2 vertebrae, 37 of 200 (18.5%) L3 vertebrae, 32 of 200 (16.0%) L4 vertebrae, and 25 of 200 (12.5%) L5 vertebrae. The lumbar MRI was conducted in our institution (“in-house”) in 99 of 200 patients (49.5%) and in another institution (“outside”, 11 different institutions) in 101 of 200 patients (50.5%). One hundred of 200 (50.0%) MR examinations were performed with scanners of Siemens Healthcare, 44 of 200 examinations (22.0%) with scanners of GE Healthcare systems, and 56 of 200 (28.0%) with scanners of Philips Healthcare. One hundred six of 200 examinations (53.0%) were conducted on 1.5-T MR scanners, and 94 of 200 (47.0%) were performed on 3-T scanners.

### Vertebral body measurements

**Fig. 4:**
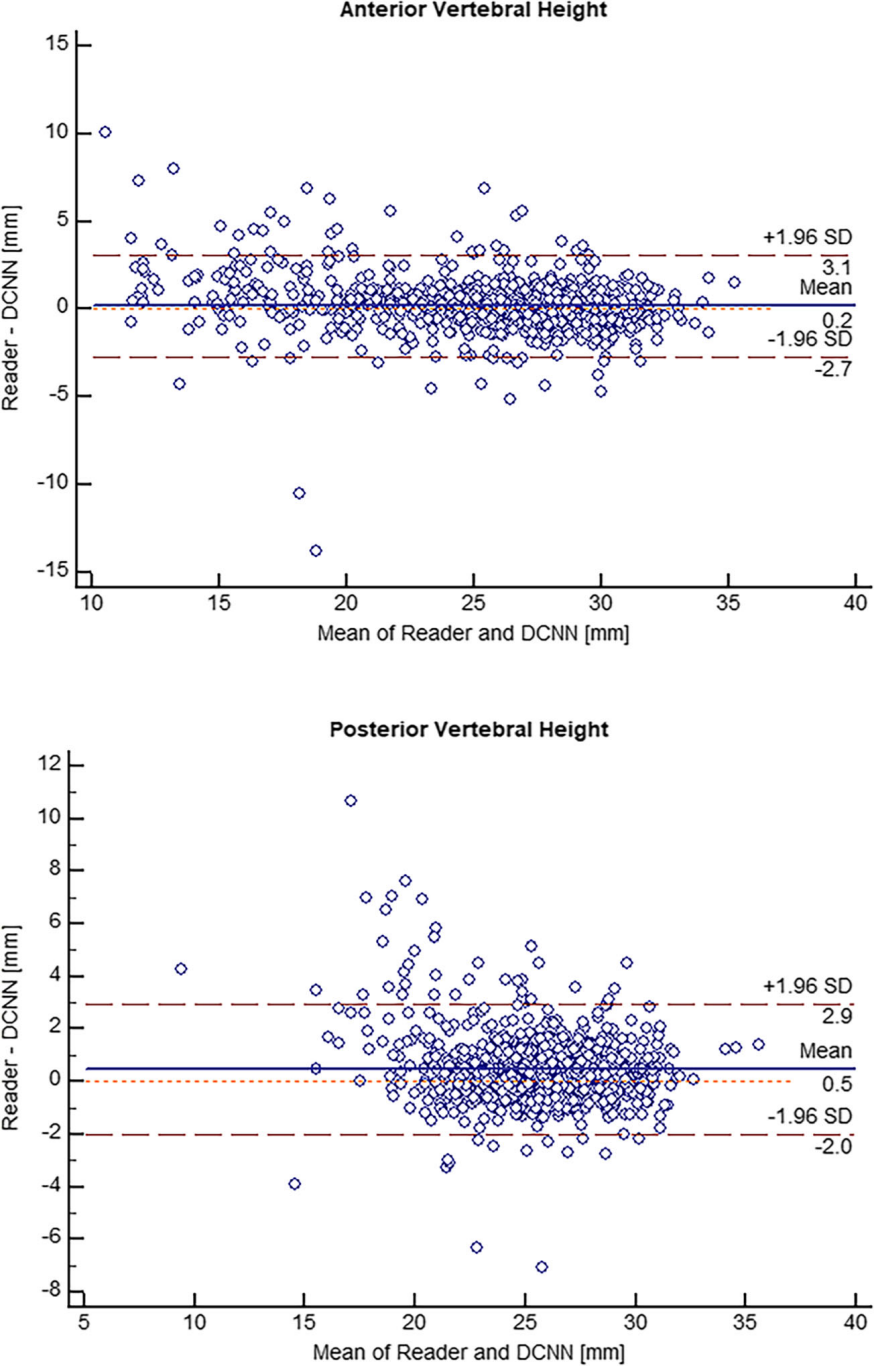
Bland-Altman plots for anterior and posterior vertebral height between measurements of the radiologists and the DCNN. Values are in millimeters. *DCNN*, deep convolutional neural network. *SD*, standard deviation.

Table 3 shows ICC values with 95% confidence intervals and kappa values between radiologists and the DCNN. Figure 4 presents the Bland-Altman plots for anterior vertebral body height (AH) and posterior vertebral body height (PH). Supplemental Figure 2 and 3 illustrate Bland-Altman plots for the maximum extent of the concavity of the superior (SC) and inferior endplate (IC) as well as vertebral angle (VA) measurement.

**Table 3:** Agreement for vertebral body measurements and fracture detection

	*R1 vs R2*	*R1 vs DCNN*	*R2 vs DCNN*
AH *ICC, 95% CI*	0.976 (0.972–0.978)	0.970 (0.966–0.974)	0.962 (0.957–0.966)
PH *ICC, 95% CI*	0.965 (0.960–0.969)	0.946 (0.939–0.952)	0.949 (0.943–0.955)
SC *ICC, 95% CI*	0.932 (0.923–0.940)	0.846 (0.826–0.864)	0.834 (0.812–0.853)
IC *ICC, 95% CI*	0.891 (0.877–0.904)	0.792 (0.765–0.816)	0.788 (0.759–0.812)
VA *ICC, 95% CI*	0.970 (0.966–0.974)	0.944 (0.936–0.950)	0.941 (0.933–0.948)
Fracture *kappa*	0.986	0.907	0.894

Interreader agreement between reader 1 (R1) and reader 2 (R2), R1 and DCNN, and R2 and DCNN, based on the individual interpretation data of each reader. ICC values with 95% CI are shown for measurements of anterior vertebral body height (AH), posterior vertebral body height (PH), the maximum extent of the concavity of the superior endplate (SC) and the inferior endplate (IC), and vertebral angle between the superior and inferior endplate (VA). The last row presents kappa values for vertebral body fracture detection

*CI* confidence interval, *DCNN* deep convolutional neural network, *ICC* intraclass correlation coefficient

Inter-reader reliability regarding AH and PH was “excellent” between reader 1 and reader 2 (ICC for AH = 0.976; ICC for PH = 0.965), between reader 1 and DCNN (ICC for AH = 0.970; ICC for PH = 0.946), as well as between reader 2 and DCNN (ICC for AH = 0.962; ICC for PH = 0.949). The agreement for SC measurement was “excellent” between reader 1 and reader 2 (ICC = 0.932), and “good” between reader 1 and DCNN (ICC = 0.846) as well as between reader 2 and DCNN (ICC = 0.834). Regarding IC measurement, the agreement was “good” for all three comparisons: reader 1 and reader 2 (ICC = 0.891), reader 1 and DCNN (ICC = 0.792), and reader 2 and DCNN (ICC = 0.788). Measurement of the VA yielded “excellent” agreement for all comparisons: ICC = 0.970 (reader 1 and reader 2), ICC = 0.944 (reader 1 and DCNN), and ICC = 0.941 (reader 2 and DCNN).

Bland-Altman analysis between the assessments of the DCNN and the average measurements of both radiologists revealed good agreement within the limit of few millimeters for height measurements: for AH, a mean difference of 0.2 mm with LoA of ± 2.9 mm; for PH, a mean difference of 0.5 mm with LoA of ± 1.5 mm; for SC, a mean difference of 0 mm with LoA of ± 2.7 mm; and for IC, a mean difference of − 0.2 mm with LoA of ± 2.0 mm. The mean difference regarding VA measurement between radiologists and the DCNN was 0° with LoA of ± 6.9° (Fig. 4 and supplemental Figure 2 and 3).

### Diagnostic performance of the facture detection algorithm

Table 4 shows the sensitivity, specificity, and accuracy with 95% confidence intervals for fracture detection of the DCNN. Table 5 presents the contingency table with numbers of true-positive, false-positive, true-negative, and false-negative classifications by the DCNN.

**Table 4:** Diagnostic accuracy of DCNN for fracture detection

	Sensitivity (95% CI)	Specificity (95% CI)	Accuracy (95% CI)
Entire cohort *n = 200*	0.941 (0.903–0.968)	0.969 (0.954–0.980)	0.962 (0.948–0.973)
L1	0.967 (0.908–0.993)	0.954 (0.895–0.985)	0.960 (0.923–0.983)
L2	0.942 (0.841–0.988)	0.919 (0.863–0.957)	0.925 (0.879–0.957)
L3	0.946 (0.818–0.993)	0.988 (0.956–0.999)	0.980 (0.950–0.995)
L4	0.906 (0.750–0.980)	0.982 (0.949–0.996)	0.970 (0.936–0.989)
L5	0.880 (0.688–0.999)	0.989 (0.959–0.999)	0.975 (0.943–0.992)
Institution			
“In-house” *n = 99*	0.957 (0.903–0.986)	0.966 (0.942–0.982)	0.964 (0.943–0.978)
“Outside” *n = 101*	0.926 (0.864–0.965)	0.971 (0.949–0.986)	0.960 (0.940–0.976)
MRI scanner			
Siemens *n = 100*	0.958 (0.904–0.986)	0.966 (0.943–0.982)	0.964 (0.944–0.979)
GE *n = 44*	0.980 (0.896–0.999)	0.959 (0.917–0.983)	0.964 (0.930–0.984)
Philips *n = 56*	0.884 (0.784–0.949)	0.981 (0.952–0.995)	0.957 (0.926–0.978)
Field strength			
1.5 T *n = 106*	0.930 (0.871–0.967)	0.978 (0.958–0.990)	0.966 (0.947–0.980)
3.0 T *n = 94*	0.955 (0.897–0.985)	0.958 (0.932–0.977)	0.957 (0.935–0.974)

Sensitivity, specificity, and accuracy (with 95% confidence intervals) are given for the entire cohort (*n* = 200), for each vertebral body (L1–5) separately, and for subgroups based on (a) the institution in which the lumbar spine MRI was acquired (“in-house” or “outside”), (b) the MRI scanner vendor (Siemens, GE, or Philips), and (c) the magnetic field strength of the MRI scanner (1.5 T or 3 T)

*CI* confidence interval, *DCNN* deep convolutional neural network

**Table 5:** Contingency table for fracture detection by the DCNN

	True positive	False positive	True negative	False negative
Entire cohort *n = 1000 vertebrae*	224	24	738	14
L1	89	5	103	3
L2	49	12	136	3
L3	35	2	161	2
L4	29	3	165	3
L5	22	2	173	3
Institution				
“In-house” *n = 495 vertebrae*	112	13	365	5
“Outside” *n = 505 vertebrae*	112	11	373	9
MRI scanner				
Siemens *n = 500 vertebrae*	113	13	369	5
GE *n = 220 vertebrae*	50	7	162	1
Philips *n = 280 vertebrae*	61	4	207	8
Field strength				
1.5 T *n = 530 vertebrae*	119	9	393	9
3.0 T *n = 470 vertebrae*	105	15	345	5

Numbers are presented for the entire cohort (*n* = 1000 vertebrae), for each vertebral body (L1–5) separately, and for subgroups based on (a) the institution in which the lumbar spine MRI was acquired (“in-house” or “outside”), (b) the MRI scanner vendor (Siemens, GE, or Philips), and (c) the magnetic field strength of the MRI scanner (1.5 T or 3.0 T)

*DCNN* deep convolutional neural network

**Fig. 5:**
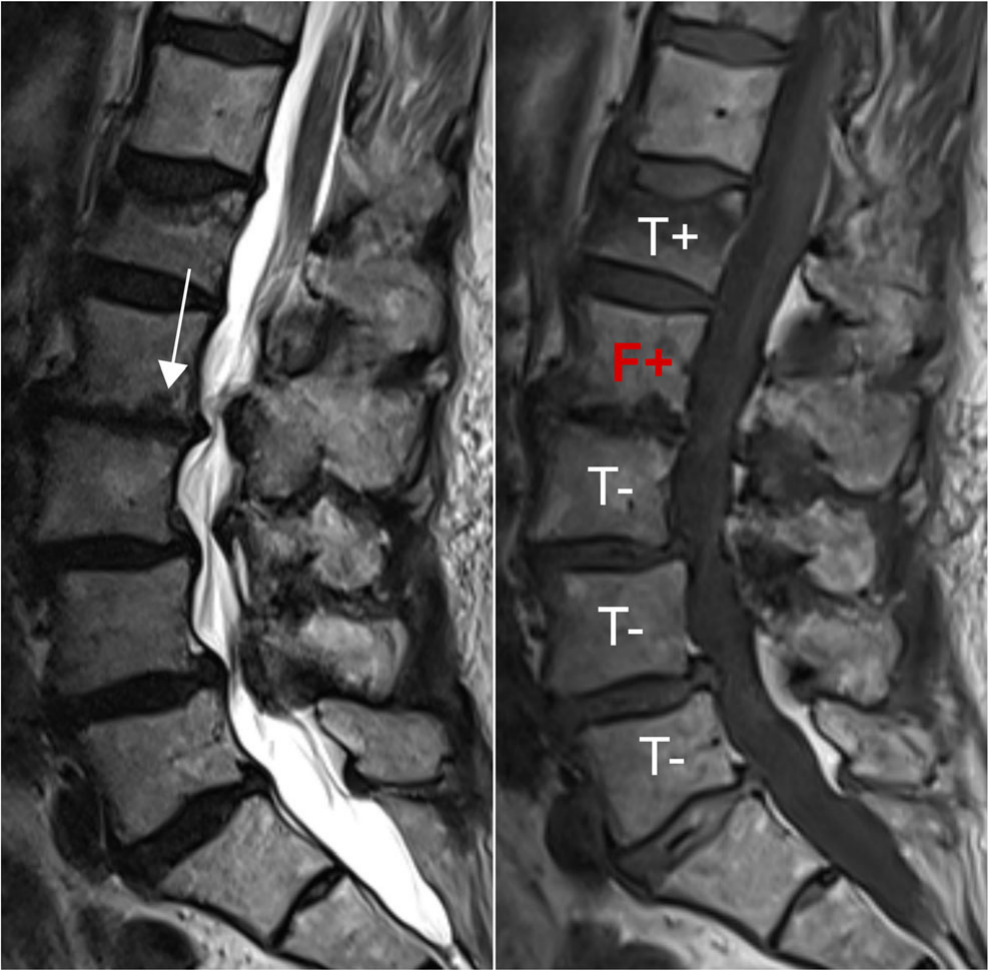
False-positive fracture classification (F+) by the DCNN. 82-year-old female patient with sagittal T2-weighted (left) and T1-weighted (right) MR images of the lumbar spine. The arrow depicts severe inferior endplate irregularities of L2 due to osteochondrosis, which may have caused the false-positive classification for vertebral body insufficiency fracture (F+) by the DCNN. The DCNN classified the L1 correctly as “fracture” (true positive: T+) and the L3, L4, and L5 correctly as “no fracture” (true negative: T−). *DCNN*, deep convolutional neural network

**Fig. 6:**
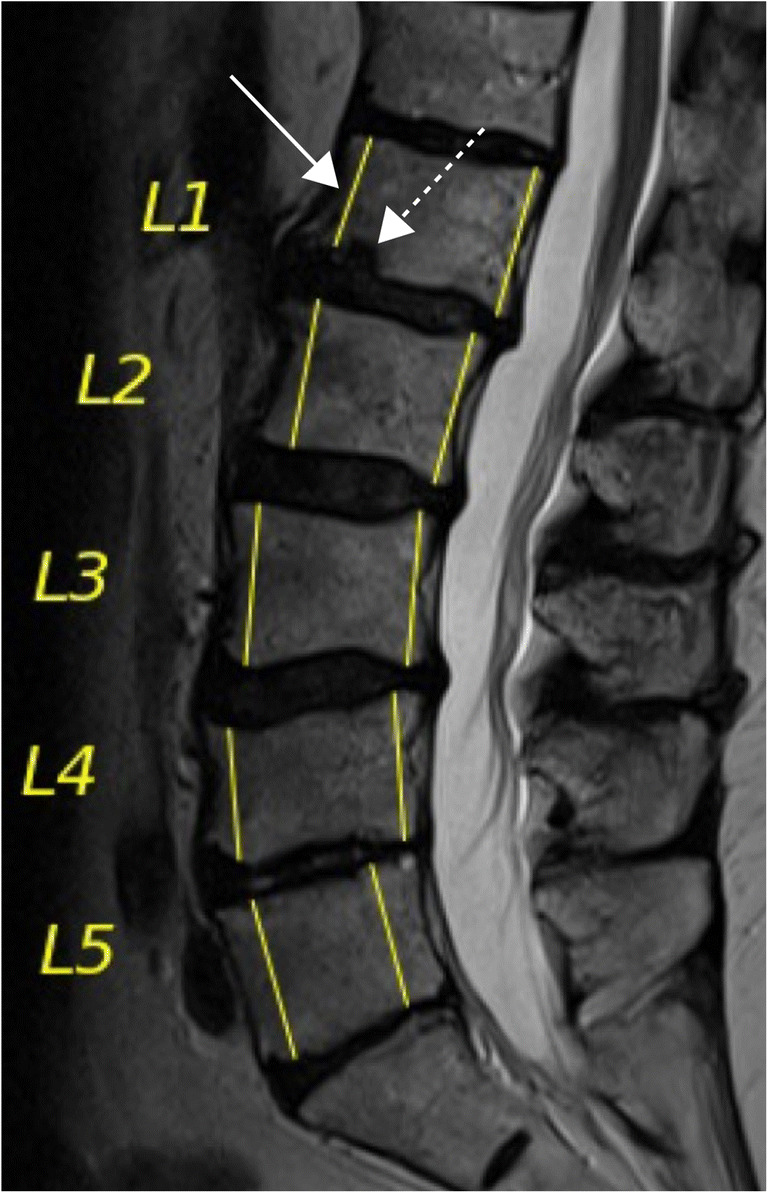
Output of the DCNN. Sagittal midline T2-weighted MR image of the lumbar spine of a 63-year-old female patient. Correct vertebral segmentation output (L1–L5) and measurements for anterior and posterior vertebral height by the DCNN are shown in yellow. The DCNN detected a reduced anterior height of L1 (arrow) based on an adjacent intraspongious herniation of the inferior endplate (dashed arrow), which may have led to a false-positive fracture classification. Measurements of L2–L5 resulted in correct negative fracture classifications. *DCNN*, deep convolutional neural network

The DCNN demonstrated an overall high diagnostic performance well above the 90^th^ percentile: sensitivity of 0.941 (0.903–0.968), specificity of 0.969 (0.954–0.980), and accuracy of 0.962 (0.948–0.973). Of the 1000 analyzed vertebral bodies, the DCNN produced 24 false-positive and 14 false-negative cases. Reviewing the 24 false positives, 11 (45.8%) had a substantial intraspongious herniation, and 6 (25.0%) had severe osteochondrosis (Figs. 5 and 6). Regarding the 14 false negatives, 8 (57.1%) had a non- or only minimally displaced fracture without significant height loss of the vertebra (Supplemental Figure 4), which may have caused the false interpretation by the DCNN.

The performance of the DCNN was independent of the origin of the MRI examination (“in-house” or “outside”) with overlapping 95% CIs (accuracy: 0.943–0.978 vs. 0.940–0.976). Furthermore, the type of the MRI scanner (vendor) had no significant influence on the diagnostic performance of the DCNN with overlapping 95% CIs for all three comparisons (accuracy: 0.944–0.979 vs. 0.930–0.984 vs. 0.926–0.978). Lastly, the DCNN performance was comparable between MRI examinations at 1.5-T and 3.0-T magnetic field strength with overlapping 95% CIs (accuracy: 0.947–0.980 vs. 0.935–0.974).

## Discussion

In this retrospective analysis, we evaluated the performance of a dedicated DCNN for MRI-based lumbar vertebral body measurements and detection of insufficiency fractures in a large patient cohort. The examinations were performed in various institutions, resulting in a large diversity of included pulse sequence protocols, which represents the vast heterogeneity of MRI examinations in radiological practices. We found that the DCNN demonstrated good to excellent agreement with radiologists for all measurements and had high diagnostic accuracy for vertebral insufficiency fractures with a sensitivity, specificity, and accuracy of 0.94, 0.97, and 0.96, respectively.

Our study is one of the first to assess the reliability of deep learning–based measurements of vertebral bodies on MRI. Recently, Suri et al showed the potential of a deep learning model to accurately measure vertebral body deformity on MRI, CT, and radiography with measurement errors around 2% [37]. Similarly, the DCNN in our study showed excellent agreement with each radiologist for measuring the anterior and posterior vertebral body height, and the vertebral angle, which was similar to the interobserver reliability between both radiologists. Insufficiency fractures are primarily a disorder of the elderly, which is the main reason for the high mean age of 75 years of our study population, and they are associated with degenerative lumbar spine disorders. Accompanied endplate irregularities and osteophytes impede the exact determination of the proper anterior and posterior endplate corner points for the morphological measurements, which might have reduced the agreement between the radiologists and the DCNN. The assessment of the extent of endplate impressions revealed a slightly lower but still good agreement between the DCNN and both radiologists. However, also the inter-radiologist agreements were lower for this assessment compared to the anterior or posterior vertebral heights. Arguably, the presence of Schmorl nodes and, to a lesser degree, degenerative endplate changes was the reason for the slight disagreement in measuring the diameter of the endplate depressions.

The DCNN detected lumbar vertebral insufficiency fractures with an overall high diagnostic accuracy. The sensitivity of 0.941, specificity of 0.969, and accuracy of 0.962 may suggest its utility as a screening tool in clinical practice. Only a few diagnostic accuracy studies have been published regarding deep learning–assisted fracture detection in the lumbar spine. A recent study demonstrated enhanced sensitivity and a tendency for improved specificity provided by AI assistance (deep learning algorithm) for fracture detection in X-ray images of the whole musculoskeletal system, analyzed by both non-radiologists and radiologists [28]. However, a subgroup analysis within that study, focusing solely on the thoracolumbar spine (*n* = 56), indicated no relevant improvement in diagnostic accuracy for fracture detection when using the AI assistance [28]. Another study showed high diagnostic accuracy for computer-aided diagnosis of lumbar vertebral body fractures on dual-energy X-ray absorptiometry (DEXA) images [29]. The authors found a sensitivity of 0.818 and a specificity of 0.974. In 2017, Burns and colleagues examined automated vertebral body compression fracture detection on computed-tomography images and found a sensitivity of 0.957, a value that is comparable to our results [30]. However, this study did not apply a deep learning–based architecture but a height compass to distinguish between fracture and no fracture [30]. The number of published studies evaluating deep learning architectures for MRI-based diagnosis of vertebral body fractures is sparse. Yabu and colleagues evaluated the performance of a DCNN algorithm to differentiate between fresh and old osteoporotic vertebral fractures based on MR images: the authors presented a sensitivity of 0.88, a specificity of 0.88, and an accuracy of 0.88 for the DCNN, which was similar to the performance of spine surgeons [33]. Yeh et al and Yoda et al recently examined the accuracy of a deep learning–based method for distinguishing benign and malignant vertebral body fractures on MRI using either sagittal T1- and T2-weighted images at 1.5 T [32] or sagittal T1-weighted and STIR images at 1.5 T or 3.0 T [34]. The results of both studies yielded comparable diagnostic accuracies for the DCNN compared to radiologists [32] and spine surgeons [32, 34]. The fractured vertebrae had to be outlined manually using a drawing box in these studies. In contrast, we detected the fractured vertebrae with our fully automated algorithm in an end-to-end fashion. Future projects may concatenate such DCNNs and may be able to achieve coverage and detection of multiple lumbar spine disorders.

Regarding fracture detection, out of 1000 vertebral bodies, our DCNN classified 24 false-positive and 14 false-negative cases. Seventeen of 24 false-positive cases revealed substantial intraspongious herniation or degenerative endplate irregularities, which may explain the misclassification.

Interestingly, with our DCNN, we did not find significant differences in the measurements or fracture detection on images acquired with different scanners or different field strengths. This emphasizes the generalizability of our findings, meaning the performance of the DCNN should achieve identical results irrespective of the MRI scanner and pulse sequence protocol used.

To succeed in daily practice, decision support tools such as the DCNN presented here may be of highest value if fully integrated into the clinical workflow of radiologists and other physicians [42]. This DCNN was implemented on a dedicated server connected to the PACS system. It received and analyzed the MR examination and returned the result to the PACS system, displaying it in PDF format next to the DICOM images.

Our study has limitations. First, our cohort represents the elderly population, as insufficiency fractures are rare in young individuals, which might limit the generalizability of our findings. However, testing and validation of the DCNN were performed in a cohort with a broad age range which makes the influence of the patient’s age on the diagnostic accuracy of the algorithm unlikely. Second, the radiologist’s fracture classification may represent an imperfect reference standard. In vertebra with only subtle changes, misclassifications may occur, especially in chronic insufficiency fractures without bone marrow edema-like changes. Using a consensus of three experienced musculoskeletal radiologists, we aimed to minimize this potential bias. Third, the diagnostic performance of the DCNN may be slightly lower in a real-life environment (when MRI with severe motion artifacts and/or scoliosis are included), as the performance in these cases may arguably be worse by the DCNN compared to radiologists.

In conclusion, for vertebral body measurements and insufficiency fracture detections on lumbar spine MRI, the diagnostic performance of our DCNN was comparable to the diagnostic performance of the musculoskeletal radiologists.

## Supplementary information

ESM 1 (DOCX 4061 kb)

## Abbreviations

CI: Confidence interval
CNN: Convolutional neural network
DCNN: Deep convolutional neural network
DL: Deep learning
ICC: Intraclass correlation coefficient
LoA: Limits of agreement

## Declarations

### Guarantor

The scientific guarantor of this publication is Benjamin Fritz, MD.

### Statistics and biometry

No complex statistical methods were necessary for this paper.

### Methodology

• retrospective

• diagnostic study/observational

• performed at one institution
