# Supplementary material for: Performance of a deep convolutional neural network for MRI-based vertebral body measurements and insufficiency fracture detection
Source: Eur Radiol. 2022 Dec 28;33(5):3188–99. doi: 10.1007/s00330-022-09354-6 (PMC10121505; doi:10.1007/s00330-022-09354-6)

**Supplemental Figures**

**Supplemental Figure 1. Vertebral Annotation.**

Sagittal T2-weighted images are shown with the respective annotation of the lower thoracic, all lumbar and the upper sacral vertebrae.


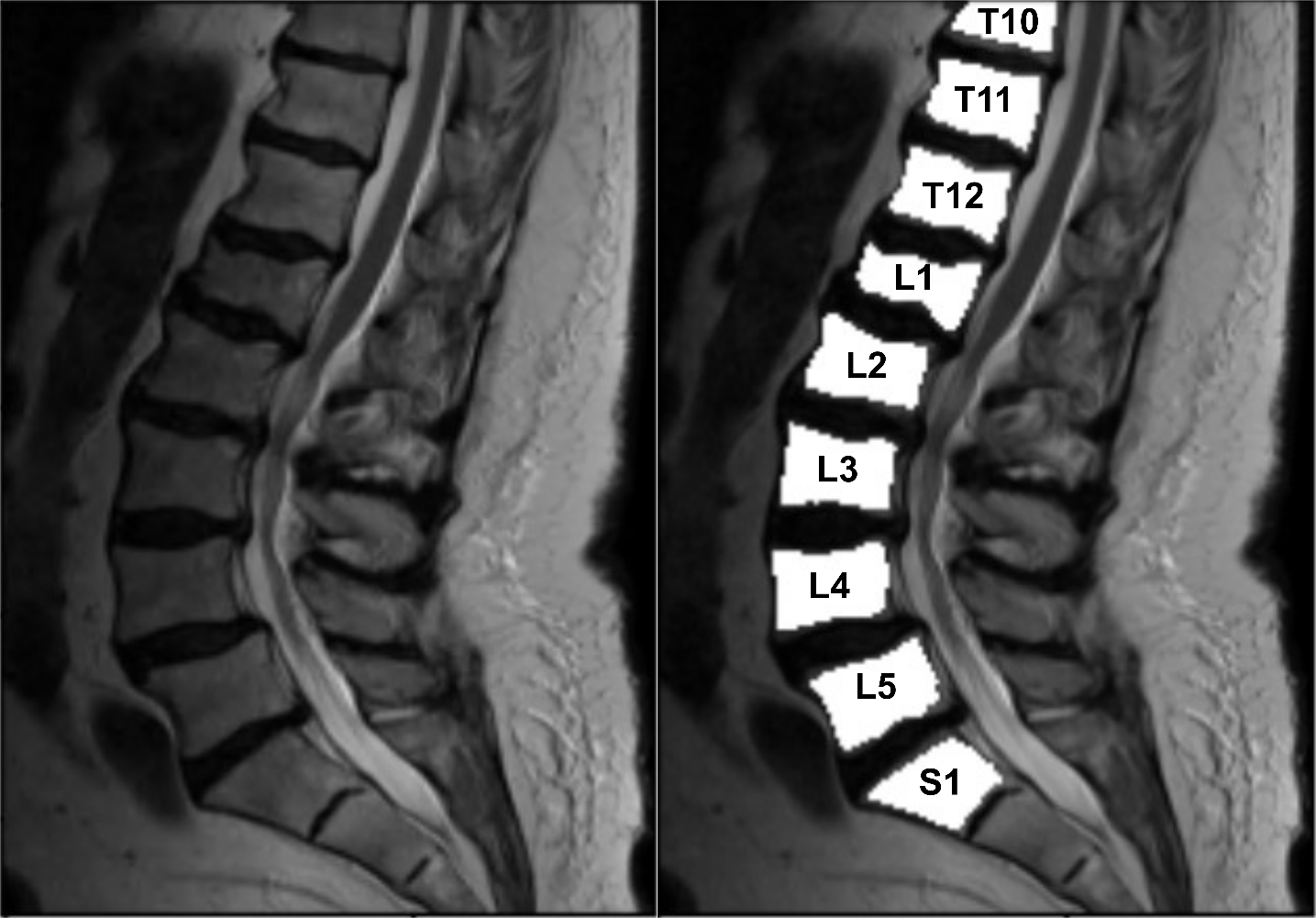


**Supplemental Figure 2:** Bland-Altmann Plots for superior and inferior endplate concavity and vertebral angle between measurements of radiologists and the DCNN. Values are in mm.

*SD*, standard deviation.

**
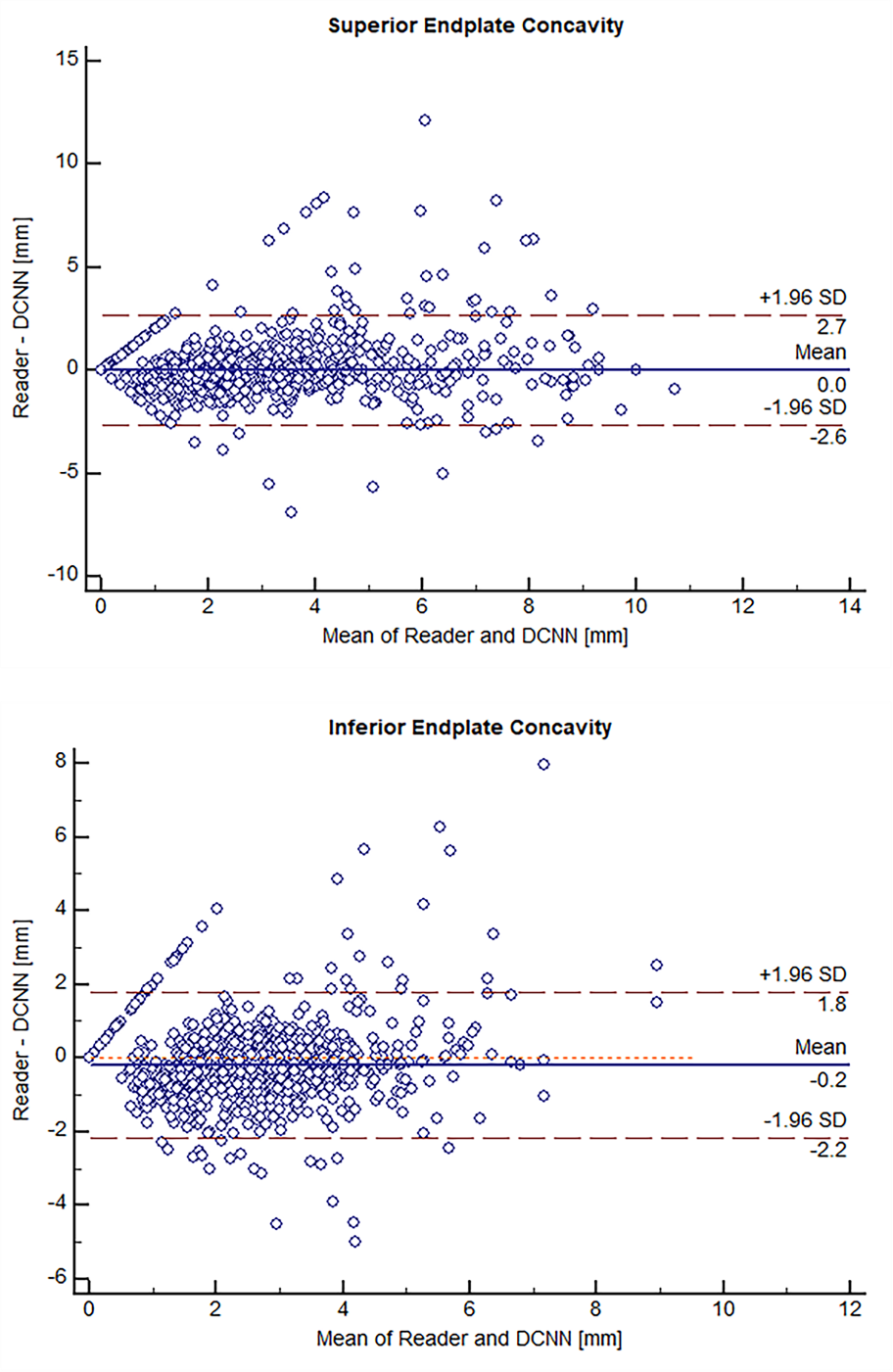
**

**Supplemental Figure 3:** Bland-Altmann Plot for vertebral angle between measurements of radiologists and the DCNN. Values are in degrees.

*SD*, standard deviation.


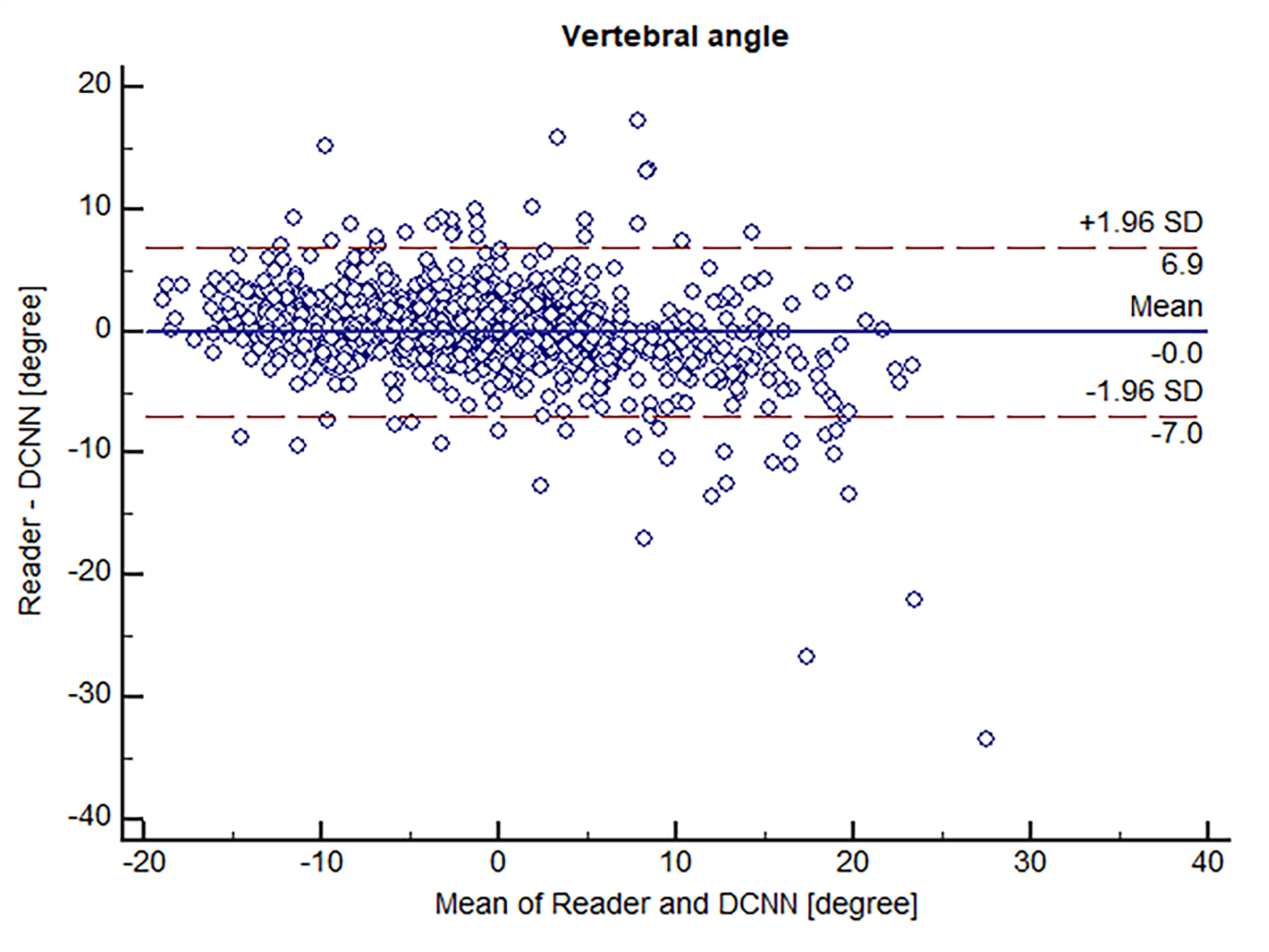


**Supplemental Figure 4. False negative fracture classification (F-) by the DCNN.**

91-year-old female patient with sagittal T2-weighted (left) and T1-weighted (right) MR images of the lumbar spine. The arrow highlights a fracture line parallel to the superior endplate of vertebral body L2. There is only a minimal superior endplate impression, which may explain the false-negative fracture classification of the vertebral body L2 (F-) by the DCNN. The DCNN correctly classified the L1 as "fracture" (true positive: T+), and the L3, L4, and L5 as "no fracture" (true negative: T-).

*DCNN*, deep convolutional neural network.


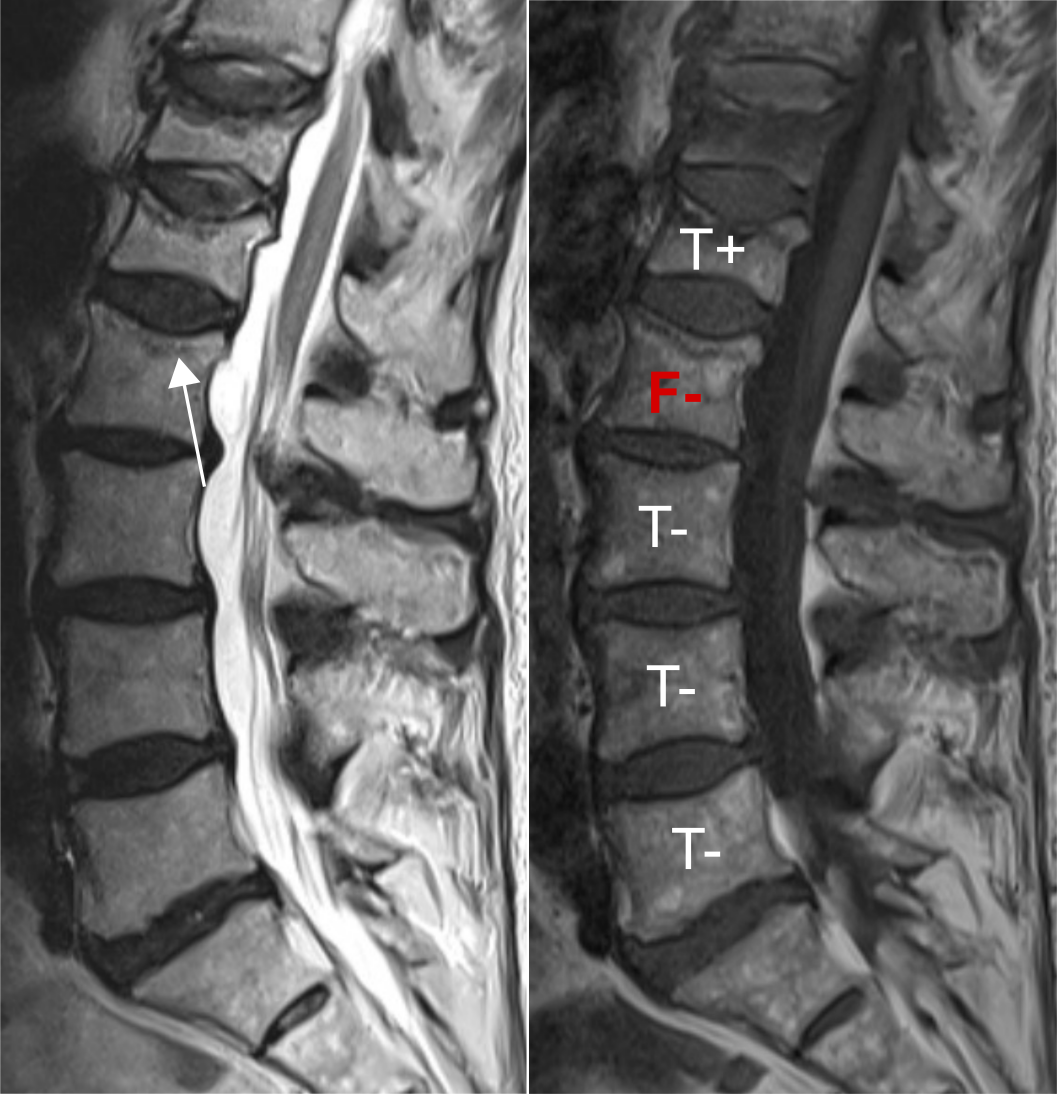

Supplement: Supplementary file 1 — (DOCX 4061 kb) [file 330_2022_9354_MOESM1_ESM.docx]
